# Practical Observations on Various Subjects Relating to Midwifery

**Published:** 1837-10

**Authors:** 


### ART. V.

1. *Practical Observations on various Subjects relating to Midwifery.* By JAMES HAMILTON, M.D. F.R.S.E., Professor of Midwifery in the University of Edinburgh. Part II.—*Edinburgh*, 1836. 8vo. pp. 428; with Plates.

2. *Observations on the artificial Dilatation of the Mouth of the Womb during Labour, and upon Instrumental Delivery, &c.* By ROBERT COLLINS, M.D., Late Master of the Dublin Lying-in Hospital.—(*Dublin Medical Journal*, March, 1837.)

3. *An Inquiry into the Management of the first Stage of Labour.* By E. W. MURPHY, M.D.—(*Dudlin Journal*, May, 1837.)

4. *Facts and Cases in Obstetric Medicine; with Observations on some of the most important Diseases incidental to Females.* By J. T. INGLEBY, Member of the Royal College of Surgeons, London; Senior Surgeon to the General Dispensary; Surgeon to the Magdalen Asylum; and Lecturer on Midwifery at the Royal School of Medicine, Birmingham. —*London*, (no date.) 8vo. pp. 296.

I. THE object of Dr. Hamilton's volume is the same as that of the first part of the "Practical Observations" of which we gave a review in our fifth Number; namely, "to put upon record those deviations from the modes of practice in the department of midwifery at present sanctioned by British and Foreign practitioners, which a long and extensive experience has led him to adopt and recommend." The first Section of the work is "on the Ordinary Management of Women after Delivery." Among other points that have been much discussed, and concerning which there has been great diversity of opinion, is the application of a roller round the abdomen of the patient, very soon after the placenta is expelled or has been removed from the uterus. Dr. H. lays much stress upon the importance and utility of the practice, and provided the roller or bandage is not applied tighter than will give a comfortable feeling of support to the patient, we think it ought never to be omitted. We are quite aware that it may, and has been urged, that many, perhaps the majority of practitioners, adopt no such precaution, or, at least, leave the application of the bandage to the nurse, who applies it in a very ineffectual manner; and that no mischief results, for the patients do well. But, on the other hand, we hear occasionally of women losing their lives after delivery from sudden syncope or unexpected hemorrhage, and we are firmly persuaded that these occasional, and we grant, rare, accidents would still less frequently occur if the bandage were speedily and efficiently applied after the uterus is emptied. We may state, at the same time, that, as far as we can judge, some of the protective advantages urged in favour of the practice by Velpeau* may be very plausible as speculations, but are by no means practically proved. Attention to the nervous system of the patient from the moment of delivery is strongly and properly advised by Dr. Hamilton. "An increased," and we may add, always suspicious, "susceptibility of puerperal women, may be easily

* Accouchemens, t. ii. 601, 2d Edit.

distinguished. The action of the heart and arteries is accelerated. The eyes and ears are affected by the slightest degree of light or noise. The patient dreams if she slumbers: when awake she is unquiet and restless." The management of such cases must depend a good deal upon the constitution of the patient, and therefore no positive rules can be laid down to guide the judgment of the practitioner. Dr. Hamilton is of opinion that, in the greater number of cases, chicken-broth, or boiled chicken, perhaps even a moderate proportion of diluted wine, should be recommended to prevent or remove this increased susceptibility of impression, instead of the farinaceous diet which in ordinary cases ought to be enjoined after delivery for the first few days. "Any attempt at suckling the infant ought to be discouraged; for, in certain constitutions, the drain of milk, independent altogether of the fatigue, is apt to occasion very serious nervous affections, such as melancholia, &c." We grant the truth of the fact that is here asserted, but we should be unwilling hastily to prevent a susceptible woman from doing that which is often one of her greatest comforts, both mentally and corporeally, namely, suckling her infant; unless, indeed, we had reason to believe, from trying the experiment, that it ought to be given up. Sleep should, if possible, be procured, and for this purpose opiates, &c. must be prescribed according to the constitution and habit of the individual. In cases of violent palpitation of the heart, Dr. H. has found musk decidedly superior to every other medicine, provided it be properly administered. Forty grains is his smallest dose. This would make the draught costly and will confine it to the rich.

Whatever might have been the faults that were once committed from the fear of properly ventilating the apartment of lying-in women, we of the present day have correct views upon the subject, scarcely keeping them at a moderate temperature: we believe that all British practitioners require cautions to guard them against the mischievous consequences of impure air and excessive heat.* In addition to the ordinary precautions to ensure the personal cleanliness of the patient after delivery, Dr. H. says that, "wherever there is any apparent febrile or irritated state of the system, the whole surface of the person should be carefully bathed, by means of a sponge, with tepid vinegar and water, at least evening and morning, for the first few days, carefully drying one part before bathing another." We are not inclined to doubt the occasional advantages of this practice, but we think its application to "any apparent febrile or irritated state of the system" is too unlimited a recommendation of it, and one that we would not advise a young practitioner to adopt.

The fear entertained by many of arresting the lochial discharge by bathing the external parts after labour with spirituous lotions, has no weight with Dr. H. He advises, as soon as the patient can bear the fatigue, that the external parts should be bathed with warm milk and water, and afterwards, as long as there is any uterine discharge, the same parts to be daily sponged with warm spirits and water; one part of proof

* Osiander's opinions upon this subject are curious, and at variance with those of British practitioners. He lays it down as one of the principal rules for lying-in women, that they should be kept in a moderate but perceptible perspiration, and denies that the sweating system formerly adopted, gave rise, as is now generally thought, to miliary fever, &c. *Handbuch der Entbindungskunst.* Band ii. 197.—Rev.

spirit to two parts of water. He has never known the lochial discharge arrested by any stimulant or astringent application to the external parts; but he has invariably found that such applications contribute in an essential degree to the restoration, both of the uterus and of the vagina, to their natural healthy condition in the unimpregnated state. For many years past he has annually seen numerous cases of chronic enlargement of the uterus, relaxation of the vagina, &c., which, he firmly believes, were the consequences of the neglect of this simple practice.

It is an established custom to give some aperient medicine the second or third day after delivery; the practice being, of course, modified according to circumstances. Dr. H. is of opinion, that, unless it be unequivocally ascertained that the bowels have been regularly cleared previous to delivery, a dose of castor-oil or aloes, combined if necessary with some narcotic, ought to be given *as soon as the woman has recovered from the shock of labour*. We admit there are cases in which this prompt exhibition of a purgative might be proper, but we can remember more in which we think that the "*as soon as*, &c." would be a little too soon.*

*Laborious Labours.* In this section of the work we arrive immediately at a point of great practical importance, which we should have discussed with Dr. Hamilton, when we noticed the first part of his "Observations," if we had not entered so much at length upon other interesting topics to which he there drew our attention. It is Dr. Hamilton's opinion that "when the pains take place, if the dilatation of the os uteri prove tedious, that is, if the continuance of strong pains for six or eight hours do not advance the dilatation to such a degree as to give reason to expect its completion within a few pains, it becomes necessary to interfere lest the patient's health should suffer." And, since the year 1800, the author has advised his pupils to secure the termination of the first stage of labour within twelve or fourteen hours from its actual commencement. And, when treating on laborious labours, "he feels it incumbent on him to declare, that, when the uterine contractions proceed regularly without decided interruption, or when the infant, after the rupture of the membranes, remains in close contact with the passages, the sufferings of the woman should almost never be allowed to continue longer than twenty-four hours, reckoning from the beginning of true labour throes." In combating the opinions and practice of so old and so experienced a practitioner as Dr. Hamilton, we readily take advantage of supporting our own views, by referring to the opposite opinions and practice of those who are most experienced; and, for this purpose, we refer to Dr. Collins's paper, the title of which we have placed at the head of this article. We most perfectly agree with Dr. Collins, that the interference recommended by Dr. Hamilton must, if *generally* adopted, be fraught with danger to the patient. It is decidedly opposed by every teacher and writer of midwifery, with the single exception of Mr. Burns, and we have always thought it our imperative duty to guard pupils against any such practice.

* That doctors differ, nobody denies: may we venture to give additional proofs of this lamentable truth. Osiander (*loc. cit.* 199,) says, "Die Wochnerin sorge nicht ängstlich für den stuhlgang und meide in den ersten Tagen alle Laxiere." Again, "Purgantia in puerperis tanquam pestis fugienda," says Baglivi. If the truth lies between these great foreign authorities and our English author, we believe that the latter is nearer the goal which all of them, no doubt, wished to arrive at.—REV.

That Dr. Hamilton and Mr. Burns may offer their interference in dilating the os uteri so dexterously and so judiciously as to inflict no injury upon patients who have the benefit of their personal attendance, we can easily believe; but we oppose as strongly as the courteous freedom of criticism will permit us, the rules laid down by Mr. Burns, and countenanced by Dr. Hamilton, for the guidance of young practitioners, whose impatience in the practice of midwifery, to cut short their patient's labour, that they may end their own, it is always so essentially necessary to guard against. It is true that Dr. Hamilton, by various remarks in both parts of his "Observations," restricts the rules of not allowing the first and second stages of labour to last longer than a limited number of hours; but still the rules remain, and are so broadly and positively laid down, that the exceptions to their application are very likely to be forgotten. We agree entirely with Dr. Collins that the number of hours a patient has been in labour ought to govern our practice much less, if it should have any weight at all, than the previous history of the patient and her actual state and condition at the time of labour. In justice, too, to Dr. Collins, we must add that Dr. Hamilton has not shown sufficient attention to his (Dr. C.'s) record of cases, as they occurred in the Dublin Lying-in Hospital, or he would not have referred to them in support of his opinions. We do not perceive that Dr. Hamilton's reply* at all blunts the force of Dr. Collins's criticism, and still less does it appear to us to show that Dr. C. has either mistaken or misstated his opinions. Dr. Collins is opposed, and very wisely we think, to artificial dilatation of the os uteri, and to any general rule which teaches the propriety of limiting the first stage of labour to any definite number of hours. Dr. Hamilton, on the other hand, quotes Mr. Burns's opinions, and eulogises his practice, and expressly tells us that Mr. B. "has zealously adopted and recommended the very treatment which he has been teaching since the year 1800." Now, Mr. Burns's doctrine is,† "that the first stage of labour ought always to be accomplished within a certain time, varying somewhat according to the constitution of the patient and the degree of pain;" and, to effect this purpose, he advises artificial dilatation of the os uteri, provided the os is lax and dilatable, and that the dilatation is gradually and gently effected during the continuance of a pain. And, again, Mr. Burns says, "that, if the pains be continuing without suspension, or an interval of some hours, and labour be going on all the time, but slowly, it is a good general rule to effect the dilatation of the os uteri within ten or twelve hours at the farthest from the commencement of regular labour."

Dr. Murphy's paper, on the *Management of the first stage of Labour*, well deserves the attention of practitioners of midwifery. After having sought for proof to determine the validity of the doctrine advocated by Drs. Hamilton and Burns, he comes to the conclusion, "1st. That no proof is given, neither do the records of the largest hospitals in Europe, nor their practice, establish, that the prolongation of the first stage of labour beyond fourteen hours, so impairs the vigour of the uterus as to become dangerous to the mother or child. 2d. That, in cases where the pains are continuing often and decided, while the os tincæ is lax, dila-

* Med. Gazette; June 10, 1837.

† Midwifery; 8th edition, p. 413.

table, and thin, the uterus hardly ever fails, unless from some obstruction in the second stage, in expelling the child with safety to both, and therefore that the practice of hurrying on the first stage of labour is totally unnecessary. 3dly. That, considering the structure of the os tincæ, how readily a derangement in the order of labour may be produced, and its liability to be inflamed from irritation, such a practice might become absolutely mischievous." In these statements we fully concur.

It has been doubted by many practitioners, whether unusual rigidity of the membranes ever retards the progress of a labour. Dr. H.'s experience, at a very early period of his life, convinced him of the fact, and he has taught his pupils, and we think very correctly, "that, if after the os uteri is completely dilated, the membranes continue entire without passing into the vagina, or if, advancing into the vagina, there be a quantity of liquor amnii interposed between them and the head of the infant, (so that the head does not enter the passage,) every labour-throe till the membranes give way is to be regarded as occasioning unnecessary and superfluous suffering." Two cases have occurred to Dr. H. where the funis was naturally so short that it became necessary to tie it, and to cut it within the vagina; consequently its length could not have exceeded six inches, and he has attended many cases where the cord was three and four times convoluted round the neck of the infant, but in none of those cases was there any impediment to delivery. In cases where the labour is protracted from deficient uterine contraction, various drugs have at various times been supposed to have the power of exciting the uterus to more efficient action. But, until lately, all confidence in such remedies was abandoned; and, in cases of ineffective pains, stimulant clysters and aperients were the chief agents relied upon. Dr. Hamilton believes that, while the excitement of the uterine action, by means of irritating clysters, may in some cases expel the infant, it far more frequently exhausts the propelling powers, and renders artificial assistance necessary. "He has never, where he had charge of the patient from the beginning, seen any case where he thought it useful to administer a stimulant enema." Our own experience has taught us to rely with more confidence upon a stimulating enema in cases of inertness of the uterus; and the fact of its ever "exhausting the propelling powers and rendering artificial assistance necessary" is new to us; and, with all due respect, we doubt it. Dr. H. is decidedly opposed to the use of the secale cornutum, but "he has only had two opportunities in practice of making a fair trial of this medicine," (p. 82.) He believes that this remedy acts in no other way than by influencing the imagination; and that, in this respect, it possesses no superiority over various other medicines. In cases where the labour-pains have been suspended, by the patient dreading the agony of the last two or three bearing pains, he has prescribed a medicine which he assured the patient would immediately bring back the pains, and hitherto he has invariably succeeded." Although he has used various medicines with this view, as camphor, ammonia, æther, &c. "the pains have generally come on within less than five minutes after the first dose; in many cases, within two minutes." If the progress of a labour is retarded by diminished uterine contraction, (denying, as he does, the efficacy of the ergot,) Dr. H. says, that the means to be adopted must be accommodated to the cause of diminished action. Some very judicious yet brief cautions are given as to the administration of opiates during labour. "There is less risk of erring in the use of the lancet than in the exhibition of opiates, for there are few cases of protracted labour where bleeding can be injurious." The danger arising from the improper use of opiates both in the latter months of pregnancy and during labour cannot be denied. We attended, for example, twenty-two years ago, a lady with her twelfth child. She had always had safe and quick labours. During the last three months of her last pregnancy, she took opiates freely. When the period of labour arrived, the uterus acted very feebly, and, after protracted suffering, the use of the forceps was indicated. She was safely delivered, but she died in a few days from low fever and peritoneal inflammation. Is it unfair to infer that, in this case, the action of the uterus was interfered with by the influence of opium? On the other hand, we do not at all doubt the frequent and striking advantages resulting from the use of the lancet in cases of protracted labour; but we must hint that the author is, in our opinion, a little incautious when he states that there are few cases where bleeding can be injurious; and we may take this opportunity of saying that Dr. Hamilton not unfrequently states his opinions too exclusively. As his authority is great, his practical doctrines and axioms should be deliberately considered, and carefully worded.

In reference to the remarks which are made at page 99 *et seq.* upon the practice in the Dublin Lying-in Hospital, under "the very able superintendence of Dr. Collins and Dr. Kennedy," we must observe that Dr. C. proves, in his "Observations," p. 46, that Dr. Hamilton has not read the work he criticises with sufficient attention, and that, consequently, many of his strictures, (to use a mild term,) are incorrect and unmerited. We will give but one example to shew how justly Dr. Collins complains of the criticisms of Dr. Hamilton. After referring to the lamented case of the Princess Charlotte, Dr. H. observes, (p. 103,) that "that melancholy case strongly shews the fallacy of a rule which appears extremely plausible," and which has been scrupulously adopted, according to the author's interpretation of the recorded cases, in the Dublin Lying-in Hospital. The rule to which he alludes is delaying interference "as long as the head of the infant advances ever so slowly, (*Dr. Collins*, p. 17)." Now, we find by reference to Dr. C.'s work, that Dr. Hamilton quotes but a part of a sentence; places a full stop when there is only a comma in the work, and thus, no doubt by some momentary but still inexcusable inadvertence, completely misrepresents the opinions of the writer. Dr. Collins's rule is thus stated: "Let it be carefully recollected that so long as the head advances *ever so slowly*, the patient's pulse continues good, the abdomen free from pain on pressure, and no obstruction to the removal of the urine, interference should not be attempted unless *the child is dead*."* We cannot wonder that Dr. Collins should ask "whether it is possible a more distorted view of his practice could be given than the *quotation* represents?" In reference to the use of the stethoscope, for the purpose of determining, during labour, whether the child is alive or dead, which Dr. Collins so properly considers a very important improvement in the practice of midwifery, Dr. Hamilton remarks, that "he cannot imagine a case of laborious labour, which had

Practical Treatise on Midwifery, p. 17.

been much protracted, where the knowledge of the state of the infant can be necessary to regulate the practice." We are much surprised at this opinion, and cannot but think it has been incautiously given. We would ask, has it never happened to Dr. Hamilton to see cases of laborious labour, where he has applied the forceps with some difficulty to himself, and even some hazard to the patient, when, if he could (and before the use of the stethoscope was applied to this point of practice, *he could not*, notwithstanding the precise and dogmatic manner in which some writers on midwifery lay down certain signs, sensations, and symptoms, as indicative of the death of the child,) have ascertained beyond the possibility of doubt that the child was dead, he would without delay have opened the head and thus have avoided the *experimental* use of the forceps. And, again, does it very unfrequently happen in the practice of midwifery, that, from the natural and very laudable horror which every man must feel to open the head of a *possibly* living child, that the sufferings of the mother are allowed to continue, in cases of difficult labours, until she is, at least, in some jeopardy, when, the means being obtained of determining the death of the child, would relieve the practitioner from great responsibility, and the mother from danger, because he would have no motive for delay, but would at once lessen the head by craniotomy. What we know of the use of the stethoscope in such cases leads us to concur entirely in the opinion given by Dr. Collins, that it is a most valuable, and much required addition to our obstetrical knowledge. That it will *always* clearly and positively determine either the life or the death of the fœtus, we do not assert.

We agree with Dr. Hamilton, that Dr. Collins restricts the use of the forceps within too narrow limits. The rules laid down by Dr. C. are *quite unobjectionable* as guides for young and inexperienced accoucheurs; but we can scarcely doubt that he himself would, in certain cases, apply the forceps where the head of the child was wholly or even nearly *in* the cavity of the pelvis, but where an ear could not be felt. This we have done several times in our own practice, and have succeeded in effecting delivery without any injury either to the mother or the child.*

Dr. H. has had great experience in the induction of premature labour. He was the first to try the effect of separating a portion of the decidua from the cervix uteri, for the purpose of bringing on premature labour without rupturing the membranes; and he shews, by referring to practical results, that many more children have been born alive after this mode of inducing labour, than when the membranes have been previously ruptured.† "With great confidence, therefore, the author can recommend this practice, in all cases where the deficiency of space, in the apertures of the pelvis, does not fall under two inches and a half." Where experience has shown that a woman has borne children of an unusual size, the author has considered that it might be proper to advise

* In our Number for July, 1836, p. 16, we have touched somewhat more at length upon Dr. Collins's opinions upon the use of the forceps.

† Velpeau, p. 413, Accouchemens, Ed. 2de, says, "Le décollement des membranes ne suffit point en effet pour mettre en jeu les contractions uterines." We rely, however, upon Dr. Hamilton's statement, and may add, perhaps for the information of M. Velpeau, that the induction of labour by this means depends greatly upon a large portion of the decidua being separated from the cervix.—REV.

this operation when there was no actual deficiency of space. Is not this a rather dangerous licence?

Upon the subject of preternatural labours, Dr. H. says, that the first object in the treatment is to determine in what manner the infant can be adapted to the passages with the greatest facility to the parent. The former, and even the prevailing, doctrine now is, that the fore part of the fœtus should be turned to the back of the mother. Dr. H., however, agrees with Baudelocque, that, "in every case where the feet are brought down, the toes should, in the progress of extraction, be turned into such a position that the belly, the breast, and the face, shall be made to pass in succession along the nearer sacro-iliac synchondrosis. After the arms are disengaged, the face can be readily turned into the hollow of the sacrum."

The early interference recommended by Dr. Hamilton in "footling cases" is opposed by Dr. Collins, and, as far as we know, by all modern writers and teachers; and we must add, that mature reflection and ample experience convince us that it is unnecessary, and, as far as the child is concerned, unsafe. Once more we must defend Denman's opinions upon the subject of spontaneous evolution of the fœtus. Whoever has been "misled," has erred from mistaking the opinions of Denman. Velpeau,* Blundell,† Gooch,‡ &c., and we infer Dr. Hamilton also, from his having, forty years ago, "raised his warning voice against practitioners being *misled* by the opinions of Dr. Denman upon this subject," must have read Dr. D.'s observations with too little attention; for a reference to the first and second papers he published upon spontaneous evolution in 1785,§—to the first edition of his Midwifery, in 1795,—and the seventh edition published by Waller, will show that he went no further than to state that the knowledge of the occasional occurrence of spontaneous evolution might be some relief to the practitioner's mind, and prevent him from having recourse to hazardous operations, in those cases where turning either cannot be effected without great danger to the patient, or where it is altogether impracticable. But, says Dr. D., in the 7th Edition of his Midwifery, "the knowledge of this fact (spontaneous evolution), however unquestionably proved, does not free us from the necessity and propriety of turning children presenting with the superior extremities, *in every case* in which that operation can be performed with safety to the mother, or give us a better chance of saving the child." And yet, not to mention Velpeau, who has doubtless quoted Denman's opinions at second-hand, Blundell says,‖ that Dr. Denman advised "that in arm presentations we should *always* confide the delivery to the natural efforts, abstaining from the introduction of the hand into the uterus;" Gooch,¶ that Dr. D. published his cases of spontaneous evolution, "together with the inference that arm presentations may be *entirely* left to nature." Such mistakes are, no doubt, accidental, but they are not the less extraordinary.

* Accouchemens, t. 2, 275.

† Practice of Obstetricy, p. 384.

‡ Midwifery, by Skinner, p. 238.

§ London Medical Journal, vol. v. By Simmons.

‖ Loc. cit.

¶ Loc. cit., as reported by Skinner.

We must be brief in our comments upon Dr. Hamilton's opinions upon the subject of Uterine Hemorrhage. Upon many points he entertains peculiar opinions, and upon some important practical ones he differs from the best authorities. He remarks, that Professor Davis errs in supposing that Puzos recommended the rupture of the membranes, where the placenta is attached to the neck or orifice of the uterus. Puzos expressly says (p. 334,) that puncturing the membranes can only be useful in the cases he described in p. 327, viz. "Le décollement de quelque portion du placenta d'avec *le fond* de la matrice." In our 6th Number we deprecated the practice advised by Dr. Davis; and, considering the danger of it in every point of view, we did not perhaps express our dissent from such a mode of treating "unavoidable hemorrhage" with sufficient decision. Dr. Hamilton thinks the use of the plug for the purpose of arresting hemorrhage in the latter months of pregnancy, whether it be unavoidable or accidental, "to be most hazardous." His great objection to the practice is," that, from the condition of the uterus after the seventh month of pregnancy, the blood discharged by the separation of the placenta, if prevented from passing per vaginam, may accumulate within the cavity of the uterus and prove fatal." True, such an occurrence "may" happen, but that it is very improbable is granted by most if not all authorities, and the *possibility* of it is denied by many: for example, Dewees,* La Chapelle,† and Legouais,‡ who states that no such accident ever occurred at La Maternité, where it is well known the field of observation is more varied and extensive than in any other institution. We could easily accumulate evidence in support of our own belief; we could just as easily shew *why* these often talked of and dreaded internal hemorrhages *during pregnancy* are much too rare to justify the practitioner in abandoning the use of the plug in very many cases of flooding during the latter months of pregnancy; and most especially in those where from rigidity of the os and cervix uteri, which we really cannot consider with Dr. H. a "bug-bear," manual interference would be very improper and very hazardous. Dr. Hamilton also disapproves of rupturing the membranes in cases of hemorrhage; but then his experience of the practice is very limited, and cannot fairly be opposed to that of Rigby, Merriman, and others, who rely upon it; for, "during the last thirty years, he has only met with two cases where he adopted it." Gooch says‖ that, in his practice, hemorrhage never continues after the rupture of the membranes. Rigby supports the practice by more than sixty cases.¶ We quite coincide with the author in opinion that the recorded cases of success from transfusion of blood in desperate cases of hemorrhage are too few to warrant any general inferences.

*Convulsions during Pregnancy, and Labour.* In common with perhaps all modern practitioners, Dr. H. strongly recommends free bleeding in most cases of this formidable disease. The extent, however, to which he carries depletion appears to us alarming. "He never directs less than fifty ounces by weight to be drawn at first, and if there be not a decided

* Midwifery, p. 422, 1st Edition.—London.

† Pratique des Accouchemens, t. ii. p. 352.

‡ Dict. des Sc. Med., t. liv., p. 323.

§ Midwifery; 8th Edition, p. 316.

‖ Loc. cit., 257. ¶ On Uterine Hemorrhage.

improvement within the hour, he advises the same quantity to be again subtracted." "Experience, too, has taught" Dr. Hamilton a lesson which we certainly have not obtained, and which appears to us to be quite repugnant to all that is known upon the subject of the different effects of large bleedings in different constitutions: viz. "in directing the first bleeding, to disregard peculiarity of constitution, for the most delicate persons require the same quantity to be subtracted at first as the most robust." We confess it would have been particularly interesting to us if Dr. Hamilton had stated, from his "experience," not merely that in the "most delicate persons" puerperal convulsions may be cured by such very large bleedings, but that they subsequently rallied from the shock the practice must have inflicted upon them; because, if, as we strongly fear, such a state of future feebleness and broken health would be produced in the "most delicate" women by so free a use of the lancet, we certainly should hesitate to adopt the practice, even upon the authority of Dr. Hamilton. Two cases occurred to us in early life, which we will briefly mention. Two young and delicate women were attacked by severe puerperal convulsions quickly after delivery of their first children. We were then fresh from the schools, and more likely perhaps to push the doctrines of our masters to an extreme, than to deviate from them. From each of these patients we took upwards of thirty ounces of blood, being guided then in the use of the lancet, as our experience has subsequently taught us we ought not to have been, by the intensity of the convulsive struggles. Both these patients recovered from the attack, for which they were thus treated; but they neither of them enjoyed good health afterwards. Their constitutions were broken; their minds were greatly depressed; and the conviction of having carried depletion too far, of not having duly considered the power of the patients to bear it, has often disturbed us, and still the subject excites in our minds much regret. We cannot but fully concur with the opinion of Dr. Collins, "that the removal of *one hundred* ounces of blood from most women in the course of an *hour* is unquestionably much calculated to injure the future health;" and we sincerely hope, in order to prevent even the assumed necessity for such treatment, that the experience of others may confirm the confidence which Dr. C. has in a mode of practice which we believe originated with him; viz. that of keeping the patient under the nauseating influence of tartar emetic *after* the first bleeding, and free purging with calomel and jalap.

The last two sections of Dr. Hamilton's work contain the author's opinions upon the subjects of Rupture of the Uterus during pregnancy and labour. In the Appendix are contained "Cases of Cicatrix in the Vagina in consequence of Laborious Labour;" "An account of a proposed Substitute for the Cæsarian Operation;" Cases of Convulsions referred to in the Work, and Letters relating to the action of the fœtal heart before birth.

II. We perfectly agree with Mr. Ingleby, that an extensive collection of faithfully recorded and well-authenticated cases affords the best means of advancing the progress of our knowledge in medicine; as, by arranging, classifying, and comparing the facts observed, we are enabled to deduce general facts or general principles. The great objection we have to many such records of cases is the dry daily detail of unimportant circumstances which writers frequently enter into, and which very few readers have patience to peruse. Mr. Ingleby eschews this fault. His cases are succinctly stated, and only those prominent features of them are noticed from which he has derived his opinions.

The first section of the work is on the subject of Puerperal Convulsions. Mr. Ingleby very correctly remarks, that, allowing for complications and variations of constitution, the more important convulsions of the puerperal state may be referred to two principal and opposite conditions of the system: either an excited or turgid state of the vessels of the brain, (often promoted by improper diet and a neglected state of the bowels during gestation,) or by *loss of blood*, as after a dangerous hemorrhage. There is also a third state, which seems more immediately dependent upon excessive sensibility of the uterine fibres, since it generally happens under an irregular and highly painful action of the uterus during its dilatation. It is curious, but it is true, that the remark of Denman, Collins, &c. is well founded; namely, that, "where the presentation is preternatural, there is little cause to dread an attack" of convulsions. Dugés, Velpeau, and others, observe that serous plethora, or the anasarca which often accompanies a first pregnancy, especially if the œdema extends to the face and upper extremities, predisposes to eclampsia. Osiander also considers a tumid condition of the hands and face as premonitory of the attack. Mr. Ingleby has seen several cases of puerperal convulsions which were succeeded by puerperal mania, and he thinks the transition might probably be the result of the large bleedings which were necessary to subdue the primary disease.

"In the treatment of sthenic convulsions, having cut short the paroxysm, our grand object should be to remove the coma, and guard against the paroxysm recurring. There are two leading indications of treatment: first, allaying vascular excitement and relieving turgidity of the blood-vessels of the brain; and, secondly, in the failure of general treatment, lessening the volume of the uterus, either by the discharge of the liquor amnii, where the case occurs antecedent to the sixth or seventh month of pregnancy, or by the entire evacuation of its contents when subsequent to those periods. As respects general treatment, *active* and *early* depletion is indispensable. Bleeding, which is borne exceedingly well, must be enforced before effusion has taken place or a permanent impression has been made upon the brain." (P. 19.)

But, adds Mr. I., we cannot define the amount of blood to be taken away; since this must correspond with the urgency of the case and the effects produced. He does not, however, advocate the extreme amount of depletion recommended by Dr. Hamilton.

"Whether general bleeding be admissible when the fits have ceased, and the comatose state has ensued, is a nice but important point to determine. Should it be undertaken, the greatest precaution must be exercised, and its effects on the circulation narrowly observed whilst the blood is flowing: it is greatly, however, to be feared that false pathological views respecting serous plethora have much restricted the depleting system. If doubt exist, it is better to practise a moderate bleeding than to neglect it; but, in protracted states of coma, and in convulsions which arise after delivery, cupping is not only the safest, but usually the most effectual method of abstracting blood." (P. 23.)

Active purging stands next in importance to bleeding. When deglutition is impeded, calomel and croton-oil are almost the only purgatives that can be given by the mouth. Purgative injections should also be employed.

"In convulsions which occur prior to the dilatation of the uterus, or with a tendency to abdominal inflammation, the tartar emetic, in quarter or half grain doses, to produce nausea, highly extolled by Collins, appears to be a most beneficial agent." (P. 23.)

In general, medicines which stimulate the uterus are inadmissible. The ergot of rye has been advised by many practitioners; and the author is not aware, nor are we, that any valid objection can be urged against its use, if the uterus and external parts are sufficiently relaxed. But, if they are not, "no practice short of the introduction of the hand could be more hurtful." But, if the case did not admit of delay, the use of the forceps would be of course preferred, presuming the circumstances were favorable for their application. Of the action of opium in these states, Mr. Ingleby has no experience, and the testimony of authors respecting it is most conflicting. We believe that opium is rarely, if ever, a proper or a safe remedy before bleeding; and that, even after depletion, it requires great discrimination, and a perfect knowledge of the previous history of the patient, as well as strict attention to the state of the brain and circulation, to determine when it is likely to be beneficial. We have no doubt that Velpeau is right in the remark that opium merits neither all the good nor all the ill that has been attributed to its use in this disease. Shaving the head, mustard cataplasms to the legs, cold to the scalp, &c., are well known and very useful auxiliaries. Dr. Graves, in his remarks on convulsive diseases, advises* that the stream of water should be small, not poured from a great height, and be discontinued the moment the fit ceases, to be again renewed on the appearance of another paroxysm. Great care should be exercised in the application of blisters. Mr. Ingleby has seen them productive of much mischief.

When general treatment fails to subdue the violence of the paroxysm, or to prevent its recurrence, then arises the question of delivery. Upon this subject Mr. Ingleby first takes a brief view of the opinions of the best authorities and then states his own.

"The want of success in delivering generally arises from one of two causes; the first—delivering too early, before the uterine orifice has undergone sufficient relaxation; the second—postponing the delivery until effusion has taken place, or a fatal impression has been made upon the brain. Previous to delivery being attempted, sufficient relaxation of the uterus must therefore be obtained by bleeding or emetic medicines in nauseating doses, purgative enemata, and perhaps the application of belladonna to its orifice, otherwise we incur the risk either of an apoplectic seizure, or a laceration of the uterus or vagina. This precaution has less regard to the degree of dilatation of the os uteri, (for the orifice is not unfrequently more or less open for many days before labour,) than to its state of softness; and if a decided impression be made upon it during the paroxysm, the sooner delivery is accomplished the better. Although the uterine office often becomes relaxed earlier than we might *a priori* infer, a moderate degree of resistance is, in every delivery, both to be expected and desired: but a forcible entry into the uterus must be discountenanced by every rational practitioner." (P. 36.)

The mode in which delivery is to be effected must depend on circumstances. If the membranes are entire, the os uteri dilated or dilatable, the vagina relaxed, and the head of the child above the brim, turning should be had recourse to. If the head is low enough in the cavity o. the pelvis, the forceps should be used. In cases of that degree of pelvic

* Dublin Journ. of Medical Science, No. II.

contraction in which the forceps could not be used with any prospect of success, the practitioner may be compelled to use the perforator. Thirty-five cases are briefly detailed, and Mr. I. adds, that, since the chapter was written, he has had additional proof of the efficacy of the tartarized antimony in the sthenic form of eclampsia, the attack taking place shortly after delivery. "The agency of this medicine (says Mr. I.) is of singular value; for, whilst it lowers inordinate action, it does not produce those distressing secondary effects which follow large bleedings." (P. 61.)

The next section treats on Malposition of the Uterus, Ovaria, Bladder, and Urethra, both in the impregnated and unimpregnated state, in connexion with retention of urine. The most important malposition to which the womb is exposed consists in its retroversion, the danger of which has been very differently estimated by different authorities.* The few first cases which occurred to Mr. Ingleby were simple in their character, and yielded to the regular employment of the catheter. In one case, attended with severe pain, which Mr. I. saw six hours after the malposition (occasioned suddenly by the restraint of company,) had taken place, although the pressure upon the urethra impeded the use of the catheter, the womb rectified its position almost instantly after the bladder was emptied. His next cases were far from manageable, and were perplexing, both on account of the development of the uterus, and the severity of the symptoms. The catheter was a palliative remedy; yet these cases ultimately did well. In one case reported to Mr. I., the event was fatal; but this "was entirely owing to the patient obstinately resisting the use of the catheter." It has been denied on very recent authority,† that a real retroversion can take place after the fourth month of gestation, and it is assumed by the writer that the instances reported by Merriman were either examples of extra-uterine gestation or posterior obliquity of the womb. But a case which forms a prominent part of Mr. I.'s essay seems to refute this opinion, and to confirm the facts advanced by Merriman.

In the treatment of Retroversio Uteri, the bladder having been relieved, attention should be immediately paid to the state of the rectum, "the degree of pressure made upon it has been known to resist even the passage of an injection." The catheter, too, should be *frequently* employed; its introduction every fourth hour is preferable to the plan of retaining the instrument in the bladder. As a marked instance of the advantage of frequently emptying the bladder, the following case is given.

"I was desired by a brother practitioner to visit a woman in consequence of the uterus remaining retroverted at the fourth month of pregnancy, notwithstanding the daily introduction of the catheter for many days. The fundus had descended almost to the anus, and the os uteri was just above the brim. At my recommendation the urine was now drawn off four times daily, instead of once; and on the third day the organ was restored to the natural position." (P. 67.)

Some cases, though few in number, will not yield to the catheter, and

* Osiander says, that Retroversion of the Uterus occurs as frequently in women who are *not* pregnant as in those who are, but that the symptoms of this malposition are much more severe in the latter. *Handbuch der Entbindungskunst Zweite Auflage.* B. 3. 114. Mr. Burns's section on this subject is excellent.—REV.

† Traité Pratique, &c.; by Boivin and Duges, p. 73.

demand the rectification of the uterus by the hand or fingers. In determining to rectify this malposition, we must be influenced not only by the duration and severity of the symptoms, the period of gestation, and the supposed dimensions of the pelvis; but also by the actual size of the uterus. The mode in which the uterus is lodged in the pelvis must also be taken into account. "It may be slightly confined, literally impacted, or it may lie comparatively unrestrained." Without advocating the expediency of the practice as a general rule, Mr. I. is satisfied that the position of the retroverted uterus may be rectified with less difficulty and danger than is usually supposed.

"From a rude attempt very much may be feared; and when gentleness and skill have failed, violence will seldom succeed. A little perseverance, however, may be necessary. Having ascertained the nature of the case, and determined upon the propriety of restoring the uterus, the bladder being emptied, an attempt to raise the fundus above the brim should be made with great care, and in the proper axis, the patient resting on her hands and knees. Bleeding and the hot bath may be premised if the resistance is considerable. By placing two fingers of the left hand in the rectum against the fundus, and two fingers of the right hand behind the symphysis pubis upon the cervix, the compound action thus obtained will usually prove successful." (P. 69.)

Dr. John Clarke* believes that a proper catheter may always be introduced. Professor Burns, too, observes, "I cannot conceive any case where a gum elastic catheter could not be introduced." But Mr. I. is correct in saying that persons of undoubted skill have sometimes failed in their attempts to reach the bladder.

"Assuming, therefore, that serious difficulties in the treatment of retroversion every now and then arise, it may be laid down, that, should the continued pressure occasion inflammation of the bladder, or render the introduction of a catheter impracticable; or should a formidable obstruction arise to the passage of the fæces; the evacuation of the liquor amnii through the os uteri, or, if this is not advisable, the puncture of the inferior part of the body of the uterus through the vagina (not the rectum,) and the immediate restoration of the uterus, will be essential to the preservation of life." (P. 74.)

A case in point is given which was attended by Mr. Baynham and the author.

"The situation of his patient, who was six months advanced in pregnancy, was in every respect desperate; and as it was impracticable to pass any instrument through the os uteri, as a last resource the uterus was punctured per rectum, the liquor amnii drawn away, and rectification then speedily effected. Recovery most fortunately took place."† (P. 75.)

For further observations on the subject of retroversion and other malpositions of the uterus, ovaria, bladder, and urethra, and for some interesting cases of obstructions in the soft parts to the progress of labour, we must refer to the work.

The fourth section treats on the Induction of Premature Labour in cases of organic disease. It is well known that in cases of pelvic deformity, the induction of premature labour at the proper period not only gives us a fair chance of saving the life of the child, which must inevitably be sacrificed if the mother were allowed to go to the full term of preg-

* Practical Essays, p. 7.

† For the details of this very interesting case, see Edin. Med. and Surg. Journal, April 1830.

nancy, but that it has also the effect of greatly mitigating the sufferings of the mother. Dr. Merriman is of opinion that the induction of premature labour by art ought to be strictly confined to those melancholy cases of distorted pelvis only for which it was originally recommended.* Dr. Ashwell† has recently proposed an extension of [the practice to cases in which tumours have formed within the uterus or in connexion with enlargements of the ovary, and also to extraneous growths in the vicinity of the uterus, which are liable to inflame during gestation, or are calculated to offer a formidable obstacle to parturition. Confessing that the cases mentioned in Dr. Ashwell's paper are deeply interesting and important, yet Mr. Ingleby thinks it questionable whether they afford sufficient data to establish as a general principle the adoption of the proposed measure in similar cases; and, for the purpose of proving that his doubts upon this point are well founded, he enters at some length into a critical enquiry of Dr. Ashwell's views. He admits that cases have arisen in which the practitioner was justified in inducing premature labour, which the strict rules laid down by Merriman would have prohibited; but he doubts, both from experience and reflection, whether Dr. Ashwell has sufficiently restricted and defended the principle he so ably and zealously advocates.

"Under any circumstances, it will be most important to consider whether the disease is likely to prove fatal; for, in sanctioning the operation, we must not only believe that such a result will happen if gestation is suffered to continue, but ought to entertain a strong conviction that the evacuation of the uterus is essential to the removal or the marked mitigation of the disease. As a matter of conscience, the concurrent testimony of several eminent midwifery practitioners, in its justification should previously be obtained." (P. 172.)

The fifth section on Laceration of the Uterus and Vagina contains many good practical remarks, and the opinions entertained by Mr. I. are illustrated by nine cases. We may take another opportunity of noticing this subject soon.

*Inversion of the Uterus.* On two occasions Mr. Ingleby has traced the occurrence of this very serious accident to unskilfulness in separating the adherent placenta. He is acquainted also with two other cases where the inversion occurred spontaneously directly on the birth of the child, but in both of them the practitioner instantly returned the organ without reference to the placenta. An instance is given of a form of inversion, which, we believe, has not been noticed before.

"I was compelled, in conjunction with another practitioner, to apply the forceps under the disadvantage of uterine inertia. After the delivery of the child there was no tendency to expel the placenta; but a portion of the mass having separated, a slight effort was made with the funis. The placenta descended considerably beyond the os internum, together with a quantity of the uterus, apparently the whole of its right side, the left not being sensibly depressed. Flooding ensued. At the moment we were rather perplexed, but the nature of the displacement became evident, and the inverted part was immediately returned, together with the placenta. The adherent portion of the mass was then separated without delay, and the case treated in the usual manner." (P. 222.)

"In treating a case of inversion, with the placenta still adhering, the rules laid down by Merriman are the best which can be followed, namely, to reverse the organ, without reference to the placenta: and in case this should be found impracticable, to peel

* Med. Chir. Trans., vol. iii. p. 142.
† Guy's Hospital Reports. Nos. 1 and 2.

away the placenta, using every precaution against the occurrence of hemorrhage, and then to return the part without delay." (P. 224.)

It is admitted by all authorities, that, the sooner we attempt to reduce the inverted uterus to its natural position, the more likely we are to succeed. Mr. Ingleby, however, very properly guards practitioners against the very common fault of being influenced too implicitly by general opinion and of despairing altogether of success, and leaving the patient to her fate when the uterus has been long inverted.* Cases are referred to in which reduction of the inverted uterus was effected many hours, and even several weeks, (*Med. Gaz.* vol. vii., 783,) after the inversion had happened. Mr. Ingleby mentions a case in which he himself succeeded in replacing the uterus eight days after inversion had taken place. It should always be remembered that, however desirable it is to restore the inverted uterus as soon as possible, the patient will be exposed to more danger by violent and obstinate attempts at reduction, than by leaving the uterus inverted. Gardien† says, that reduction is sometimes easier after several days, than when only a few hours have elapsed. When the inverted uterus is not immediately restored, it generally happens that, in a few hours, its tissue becomes engorged with blood and is thickened; that the cervix contracts and presses upon the part of the organ it embraces, so as to produce inflammation. In this state, reduction is frequently impossible, and persevering attempts are hazardous. We should wait, using in the mean time proper means to reduce the congestion and inflammation of the inverted organ, as bleeding, gentle pressure, warm fomentations, &c.; and, in all probability, by following this plan the parietes of the uterus will become softer and less sensible, and the cervix will offer less resistance to moderate and safe efforts at reduction. Mr. Ingleby touches but briefly upon part of the subject which is of importance; namely, the propriety of extirpating the uterus, when it is inverted and cannot be replaced. We perfectly agree with Mr. Burns,‡ that the trial of the extirpation of the uterus is to be preferred to leaving the patient to certain death, but we believe with Murat§ that, unless ulceration or sloughing of the uterus takes place, this formidable operation is not indicated. It is worthy of remembrance, that Dewees|| mentions a case of inverted uterus, in which reduction was impossible, from the stricture of the contracted mouth of the organ. The patient was apparently in a dying state. It occurred to him that he might take off the stricture by inverting the uterus completely. In this attempt he succeeded, and the woman was almost instantly relieved from the anxiety and faintness she had before experienced. No hope of recovery, however, was entertained; but she *did* recover, without another alarming or troublesome symptom.

In the last section, the Signs and Symptoms of Pregnancy are generally described; their obscure and deceptive characters; their compli-

* Upon this very important practical subject we refer our readers to Gardien, vol. iii. 137; and Dict. des Sc. Med., t. xlvii., 465. In both works several cases are related where long standing inversion of the uterus was restored by art, and some in which nature effected the spontaneous reduction.—Rev.

† Loc. cit. 313.

‡ Midwifery, 8th Ed. 521.

§ Dict. des Sc. Med., t. xlvii., 495.

|| Midwifery, p. 539.

cation with disease; and the signs which denote the extinction of life in the fœtus: but this subject we have discussed so much at length in another part of the present Number, that we must pass it here without further notice.

It is not from mere compliment, but from a sense of justice to the author, that we conclude our notice of his work by saying that it is very creditable to his industry and judgment. His experience upon the subjects he discusses is evidently great; and his practical inferences are cautiously drawn, and generally supported by a reference to the best authorities.

---

### ART. VII.

*Untersuchungen zur Physiologie und Pathologie.* Von Dr. FRIEDRICH NASSE und DR. HERMANN NASSE. Erstes, Zweites und drittes Heft. —*Bonn*, 1835-6.

*Physiological and Pathological Researches.* By DR. FREDERICK NASSE and DR. HERMANN NASSE. Parts I. II. and III.—*Bonn*, 1835-6. 8vo. pp. 486.

